# Methodologies to assess community-based animal health workers curricula and training programs: a mini review

**DOI:** 10.3389/fvets.2025.1655253

**Published:** 2026-01-12

**Authors:** Alice Matos, Armando E. Hoet, Andrea L. Bessler, Amanda M. Berrian

**Affiliations:** 1 Department of Veterinary Preventive Medicine, College of Veterinary Medicine, The Ohio State University, Columbus, OH, United States; 2 Veterinary Public Health Program, College of Public Health, The Ohio State University, Columbus, OH, United States

**Keywords:** community-based animal health worker, competencies, curriculum, review, training assessment methods

## Abstract

**Introduction:**

Well-trained and supervised community-based animal health workers (CAHW) are key contributors to the veterinary workforce with significant potential to increase access to veterinary care for underserved communities. Recognizing their value, the World Organisation for Animal Health (WOAH) recently published the Competency and Curriculum Guidelines for CAHWs to help harmonize their training within and across countries. This recent advancement must be accompanied by a methodology for curriculum and training assessment to determine whether existing CAHW training programs meet the established recommendations.

**Methods:**

Using PRISMA guidelines, a literature review was conducted to explore the current assessment practices in CAHW training programs, aiming at guiding the development of a curriculum assessment tool. The articles were thoroughly reviewed, and relevant information was extracted and analyzed using the Kirkpatrick Model for training evaluation and the competency-based education (CBE) framework.

**Results:**

Of the 203 publications screened, seven met the selection criteria and were included in the review. Most of the authors utilized multiple methods such as interviews, surveys, and direct observation to assess different training effectiveness levels (reaction, learning, behavior, and/or results) and competency types (knowledge, skills, and/or abilities). Conversational methods of assessment were preferred, and both learners (CAHWs) and their clients (i.e., livestock owners) were frequently engaged in the process.

**Conclusion:**

This review highlights the need to develop and implement standardized curriculum and training assessments to complement the recent WOAH guidelines and, ultimately, advance CAHWs’ proficiency and animal healthcare delivery.

## 1 Introduction

Limited access to veterinary services significantly impacts both animal and public health, especially in rural and remote areas worldwide where service availability is even lower (1). Addressing this issue requires ensuring adequate education and training for key stakeholders of the veterinary workforce, particularly community-based animal health workers (CAHW), who are essential contributors to a robust One Health workforce along with veterinarians and veterinary paraprofessionals (VPP) (2).

A CAHW, also referred to as village animal health worker (1, 3, 4), is “a person selected from or by their own community and provided with short, initial or recurring vocational training to perform basic animal health and animal husbandry-related services, in line with national animal welfare standards. CAHWs operate on a fee-for-service basis or some other means, are accountable to a registered veterinarian, a registered veterinary paraprofessional or an appropriate official and are active in their community” (5). Mostly found in low- and middle-income countries (LMIC) of Africa and Asia, their functions might include providing basic veterinary care, organizing vaccination campaigns, advising livestock keepers on husbandry practices, diagnostic sample collection, assisting in disease reporting and control, and conducting food inspection (1, 4). Through these functions, CAHWs are valuable contributors to a One Health workforce, particularly at the community level, raising awareness of intersectional health problems and applying comprehensive solutions to prevent zoonotic diseases, promote food security, and ensure sustainable production management practices that preserve the health of animals and the people who depend on them.

While there are several entities dedicated to training CAHWs, from non-governmental organizations (e.g., Agronomes et Vétérinaires Sans Frontières (AVSF), Action Contre la Faim (ACF), Heifer International, International Committee of the Red Cross (ICRC)) to intergovernmental organizations (e.g., Food and Agriculture Organization of the United Nations (FAO), United Nations Children’s Fund (UNICEF), African Union Inter-African Bureau for Animal Resources (AU-IBAR)) and government agencies (e.g., Agriculture Skill Council of India, Government of Chad), there is little consensus regarding the logistical structure and content of such trainings. For example, training durations can vary significantly, from a few days to close to 1 year in total length (1, 3). Similarly, neither trainee profiles nor cohort sizes seem to be standardized between trainings; trainees have different baseline experience and levels of literacy, creating variable dynamics within and among training programs (1, 3). Instructional approaches also add variability, with varying time spent utilizing theoretical-based pedagogy vs. implementing hands-on activities (1). The lack of agreement and coordination between training programs may limit the effectiveness of CAHWs and exacerbate existing dysfunction in animal health services (1).

Although standardizing such trainings is complex, the recognition of their important contribution to the veterinary and One Health workforce led to the development of *Competency and Curriculum Guidelines for CAHWs* by the World Organization for Animal Health (WOAH) (5). With the availability of these Guidelines, it is imperative to consider curriculum and training assessment methodologies to allow existing CAHW trainings to determine whether their content meets the established recommendations. Moreover, regular training assessments are needed to ensure that competencies are effectively and consistently transferred to CAHWs. Only by supplementing the guidelines with a framework for curriculum and training assessment is it possible to fully support the preparation of proficient CAHWs who can strengthen veterinary services and expand One Health efforts.

To contribute to the development of such a framework, a literature review was conducted to offer a succinct summary of current assessment practices in CAHW training systems. The present article highlights common practices and gaps identified to guide the creation of an assessment tool for CAHW training programs, helping organizations and regulatory bodies to optimize CAHWs’ potential.

## 2 Methods

Publications were gathered from multiple databases, namely PubMed, CABI: Cab abstracts, CABI: Global Health, Scopus, CGIAR, and Google Scholar, using a modified PICO question, with no comparison (C) term, as follows:

(P) Population: “community-based animal health worker” OR “Village animal health worker” OR “CAHW” OR “VAHW” OR “para-veterinarian” OR “basic veterinary worker” OR “village veterinary promoter” OR “village livestock promoter” OR “village vaccinator” OR “livestock correspondent” OR “barefoot vet” AND.

(I) Intervention: “training” AND (“assessment” OR “evaluation”) AND.

(O) Outcome: (“competencies” OR “competency” OR “proficiency” OR “knowledge” OR “skills” OR “learning” OR “reaction” OR “satisfaction” OR “behavior” OR “behaviour” OR “results” OR “impact”). Additionally, we searched for literature produced by organizations that train or work with CAHWs including WOAH, VSF International, FAO, African Union, Heifer international, and Global Alliance for Livestock Veterinary Medicines (GALVmed) by reviewing available public reports and resources. No restrictions were applied for year of publication and geographic location, but only published documents written in English were considered.

The present research followed PRISMA guidelines (6) and utilized the online tool Covidence systematic review software, Veritas Health Innovation, Melbourne, Australia, to facilitate collaboration among the researchers (AM, AB). Initially, all results were screened by reading the abstracts or executive summaries, and papers not related to veterinary workforce development were excluded. Papers that received “yes” from both reviewers and those that had conflicting votes on the screening step were selected for full review to determine their eligibility. At full review stage, reviewers included only studies focusing on training for CAHWs in which assessment of the curriculum or training was performed. Papers solely addressing project operational processes (design, implementation, and evaluation), characteristics or sustainability of CAHWs systems, service capacity, value chain, and knowledge or practices of livestock producers were excluded. After creating a preliminary list of eligible papers, relevant articles cited in each included study and other publications by the same authors were screened to ensure comprehensive coverage. The new papers from the backward “snowball” search (7) underwent full review using the inclusion and exclusion criteria previously described.

All studies deemed eligible were thoroughly reviewed, and relevant information was extracted into an Excel file for subsequent organization and synthesis. Papers were scrutinized to collect information systematically, including the focus of the assessment (i.e., curriculum or training) and training effectiveness level according to the Kirkpatrick Model (a framework for training evaluation that considers four levels - reaction, learning, behavior, and results) (8), based on reviewers’ interpretation. When sufficient information was available, data collection methods were analyzed using a competency-based education (CBE) framework to identify which types of competencies, as defined in the Guidelines (knowledge, skill, and personal, social, and methodological abilities) (5), were assessed with each method. Other extracted information included the stakeholders involved and the use of results to improve training.

## 3 Results

**Figure 1:**
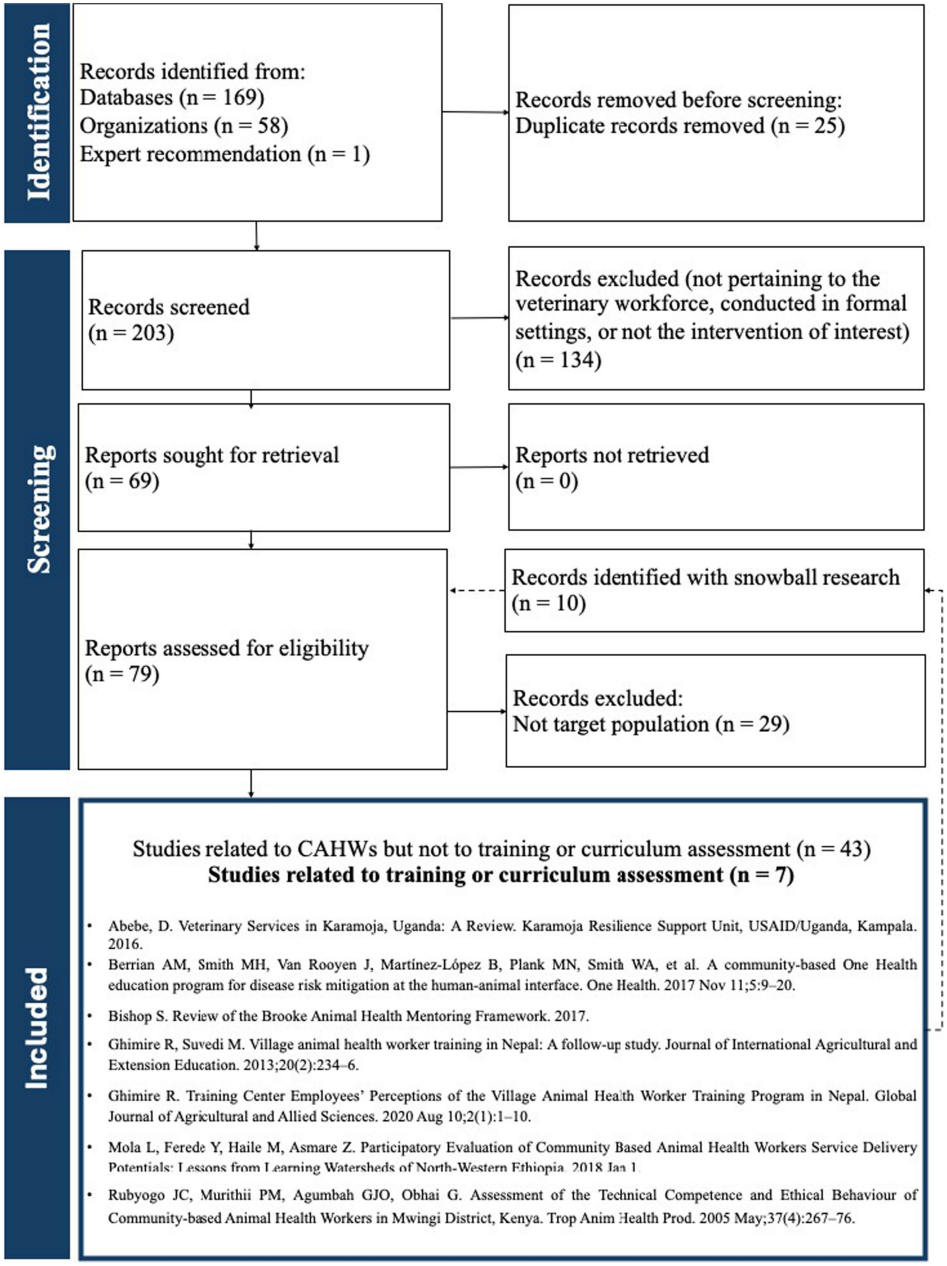
PRISMA flow chart illustrating selection of literature for inclusion in mini review.

The flow chart (Figure 1) outlines the review process and summarizes the results. From the primary research, 213 documents were screened, of which 79 were considered for full review.

Of those, 31 did not cover CAHWs curriculum or training program assessment, instead most were about services capacity (*n* = 18) or project operational procedures (*n* = 13). Additionally, 22 did not concern CAHWs, but rather VPPs, farm workers, livestock producers, or community health workers (CHWs), and 15 were general information on CAHWs systems or had considerable missing information. Importantly, four curriculum or training guidelines were found in the research process. Ultimately, seven papers met the inclusion criteria and are scrutinized in this review.

### 3.1 Overview of included papers

Abebe (9) assessed the veterinary services in Karamoja, Uganda, by examining the different animal health service providers according to their accessibility, availability, affordability, quality and acceptability. Additionally, the authors investigated CAHWs’ competence as a reflection of their training and training curriculum (9).

Berrian et al. (10) described the implementation of a four-week zoonotic disease and biosecurity program, the One Health Training and Leadership (OHTL) initiative in South Africa. The curriculum was specifically developed for this program, which included training of community-based facilitators who went on to teach village residents the concepts and practices. Residents included community leaders, cattle owners, traditional healers, healthcare workers, and teachers. The authors aimed to assess whether the program improved the professional skills and One Health knowledge of the OHTL facilitators, as well as the knowledge and skills of the participants (10).

Bishop examined Brooke’s competency framework (11), which is employed to assess and monitor the competencies of animal health practitioners, including CAHWs. The study highlights the curriculum as a critical element of the assessment framework and provides a comprehensive view of how the training is structured and evaluated. Notably, the Brooke training is mentorship-based, facilitating formative assessments through ongoing support (12).

Ghimire and Suvedi evaluated the effectiveness of the Village Animal Health Worker (VAHW) training program in Nepal, which has been in place for several years at a training center (13). Later, Ghimire investigated the perceptions of training center employees regarding the VAHW training program, offering insights into the training process and its implementation (14).

Mola et al. (15) presented a community animal health worker (CAHW) project in North-Western Ethiopia. The training manual used for the 21-day training was developed based on guidelines from Ethiopia and Kenya. While the primary aim of the article was to evaluate the effectiveness of the primary animal health services delivered by CAHWs, the authors also detailed the training and assessment procedures associated with the program (15).

Rubyogo et al. (16) explored a CAHW project in Mwingi District, Kenya. Although the authors focused on investigating the sustainability of CAHW services in this district, the study provides valuable insights into the 14-day training program and the associated training assessment (16).

### 3.2 Assessment practices

While all the authors focused on assessing training effectiveness, the level assessed and methods utilized differed. Table 1 summarizes the key training assessment methods featured in the included papers, along with a description of the Kirkpatrick Levels (8) to clarify their connection to learners’ progression. This serves as a foundation and framework for the analysis.

**Table 1:** Training assessment methods described by each author, organized by Kirkpatrick’s model evaluation levels.

Authors	Reaction^1^	Learning^2^	Behavior^3^	Results^4^	Levels of evaluation according to the Kirkpatrick Model
Abebe (9)			Semi-structured interview Observation	Semi-structured Interview Focus group discussion Observation Matrix scoring	Assessment methods
Berrian et al. (10)		Pre-/post-test Retrospective self-report Focus group discussion	Semi-structured interview		
Bishop (12)		Observation with rubric	Observation with rubric		
Ghimere and Suvedi (13)	Survey	Survey		Survey	
Ghimire (14)	Semi-structured interview (group)				
Mola et al. (15)		Post-training assessment (method not specified)		Survey Case book records	
Rubyogo et al. (16)		Test (No details)	Structured interview Direct inspection of material Observation Drug use records	Focus group discussion Interviews	

Four Levels of Evaluation According to the Kirkpatrick Model (8): ^1^*Reaction* “measures the degree to which participants find the program or initiative favorable, engaging and relevant to them” (e.g., assessment of CAHWs’ satisfaction with training). ^2^*Learning* “measures the degree to which participants acquire the intended knowledge, skills, attitude, confidence, and commitment based on their participation in the program” (e.g., assessment of CAHWs’ knowledge immediately after training). ^3^*Behavior* “measures the degree to which participants apply what they learned during the program when they are back in their environment” (e.g., assessment of how CAHWs conduct a particular task long after training completion). ^4^*Results* “measures the degree to which targeted organizational outcomes occur as a result of the initiative and subsequent support and accountability package” (e.g., assessment of animal disease prevalence and mortality rates).

Apart from Ghimere (14), all papers assessed different evaluation levels. Regarding the types of competencies, knowledge was assessed using tests, surveys, semi-structured interviews, and focus group discussions (FGD). Skills and attitude were assessed via direct observation, review of records, and inspection of materials. Self-reports on perceived competence were also used to assess knowledge and skills (10, 13). All investigators collected quantitative and qualitative data. Most investigators prioritized the use of conversational methods for data collection (e.g., interviews and FGD). However, the surveys implemented by Ghimire and Suvedi (13) and Ghimire (14) and the pre−/post-test and self-report administered by Berrian et al. (10) were in writing.

Assessments were conducted by research teams, except for Brooke’s tool which was administered by the mentor. When assessing learning and behavior levels, CAHWs were the primary respondents. Livestock owners were also a valuable source of information, particularly for assessing the results level. Another relevant source of information were the trainers (12), the supervisors (15, 16), and existing reports of CAHWs services (15, 16) Rubyogo et al. (16) used Animal Health Assistants, the lowest level of animal health service providers acceptable to the critics of CAHWs systems, as a control for comparison of CAHWs knowledge.

The marking scheme utilized by Rubyogo et al. (16) in the structured interview for knowledge assessment considered CAHWs’ language capacity, but no information was given on how. Another consideration about language is that instruments were translated to the local language (10, 13, 14). Moreover, translators were used during data collection by Berrian et al. (10) and Rubyogo et al. (16).

Assessment instruments were reviewed by experts (13), students and educators (14), external reviewers (12), or pre-tested by a small sample from the respondent group (16). Adding to the validity considerations, Berrian et al. (10) used FGD to triangulate learning assessment results. Reliability coefficient (Cronbach alpha) was calculated *post hoc* by Ghimire to verify consistency across statements’ interpretation (14).

While none of the authors explicitly aimed at assessing the curriculum, Rubyogo et al. designed the assessment based on the training curriculum, and Berrian et al. used the results to inform training improvements.

## 4 Discussion

This mini review highlights a significant research gap that must be addressed to standardize and advance training aimed at optimizing the efficiency and role of CAHWs within the One Health workforce. While the lack of a harmonized technical language across papers was a major challenge in this review, the authors used well-described frameworks to classify and analyze the assessments, allowing for comparison across very diverse papers.

The assessment of level 1 (reaction) alone (14) provides little information about the effectiveness of the training; however, when combined with other levels of assessment, reaction may help identify the root causes of learning gaps as satisfaction (or dissatisfaction) with the training methods and approaches may affect learning outcomes. Assessing knowledge long after training (13) does not reflect learning from the training, as it can be associated with a high risk of recall and maturation biases. In this paper, the authors used a single survey at one point in time to assess participants’ reaction, learning, and results levels. However, given the time elapsed since the training, only results level could be accurately assessed (8).

To assess the learning level, tests should be conducted immediately after training (15, 16) or, ideally, using a pre-/post-test (10) to assess participants’ knowledge gain. Most assessments were classified as behavior level assessments since they aimed at measuring the extent to which learned concepts were being applied in the field. However, because of the large time span between training completion and assessment, with some authors performing the assessment more than 2 years after training completion, we cannot be confident that CAHWs’ performance can be attributed to the training itself, but rather to experience gained over the practice years, being prone to maturation bias. To allow for the transfer of skills and knowledge into actual performance, behavior should be measured at least 90 days after training, but the longer the duration between training and the assessment, the higher the chances for biased results, impacting the validity of the assessment. For example, Bishop recommended using their observation-based assessment tool at least once every 3 months, but as frequently as possible (12). Despite being a resource-intensive approach that may not be feasible for most CAHW training organizations, ensuring timely assessment (e.g., during training and/or up to 90 days after completion to assess learning) and increasing the frequency is strongly recommended. Lastly, although assessing results is associated with high training transfer because it holds participants accountable for applying the training (17) and is useful to show the importance of training CAHWs (13, 15), focusing solely on long-term results provides limited insight into the training itself.

Ideally, assessments should cover all four levels, allowing to build a comprehensive narrative from immediate reaction and learning through on-the-job application and eventual results. If that is not feasible, priority should be given to learning and behavior levels. Learning is directly tied to the training and its curriculum, with minimal risk of bias, and behavior assessments have been linked with higher training transfer (17).

Importantly, most authors utilized multiple assessment methods and data sources enabling triangulation of results. This approach helps mitigate inherent limitations of individual methods, such as recall bias linked to self-assessment surveys.

Berrian et al. (10) suggested that differences in performance on written assessments across education levels may be due to unfamiliarity with the assessment format, limited literacy, or translation errors. While literacy and education level do not necessarily hinder strong performance (16), Berrian et al. (10) findings highlight the importance of designing assessment tools that take literacy into consideration to avoid misleading results.

The overall lack of papers on CAHW curriculum assessment may be due to the absence of national guidelines in some countries and the fact that, until recently, no international guidelines were available for comparison. The general lack of assessments might be due to limited resources and a lack of recognition of their importance. Since most assessments were conducted by a research team, it suggests they were one-time evaluations rather than iterative ones. This is problematic because organizations that frequently evaluate their training programs experience higher rates of training transfer (17). Furthermore, the lack of articles describing the implementation of subsequent trainings with revised curricula makes it challenging to determine the changes that lead to better training outcomes. As the demand for proven impact grows, as noted by Ghimere and Suvedi (13), regular assessments become increasingly important and sharing results can highly benefit other organizations.

The authors acknowledge the heterogeneity of the papers included in the present review. Other limitations include that only English and published research was included, and that some of those included were mostly descriptive, missing relevant elements to assess their rigor. Despite that, this review enabled several key takeaways that appear to be important to increase the validity and feasibility of curriculum and training assessment in the CAHWs settings, such as:

- Assessment modality may include conversational methods like interviews and FGD to better accommodate different literacy levels and collect comprehensive, qualitative insights of the learners’ experience.
- Trainers, CAHWs, supervisors, and livestock owners can serve as primary assessors to capture multiple perspectives gained from observation and direct interaction with the learners.
- Assessment instruments need to be administered in the local languages to avoid misleading results.
- Multiple assessment methods should be used to mitigate limitations inherent to individual methods and to cover different types of competencies and levels of assessment.

It is also important to consider the use of well-described frameworks (e.g., Kirkpatrick, Kaufman and Keller’s Five Levels of Evaluation, CIRO Model) to improve the validity of assessment results. Their use will facilitate the analysis across curricula and training programs, enabling organizations to easily exchange lessons learned. In choosing a framework, one should consider their limitations; for example, Kirkpatrick fails to capture individual and contextual factors like motivation and support systems, respectively (18).

Future research could be enriched through inclusion of unpublished training manuals, project reports, and non-English literature to better capture the breadth of CAHW programs. As more research becomes available on this topic, a meta-analysis could be beneficial to further support the choice of assessment methods. Despite the limitations, to the best of our knowledge, this is the first review conducted on this topic, being an important step toward the development of a systematic methodology to assess CAHWs’ training and training curricula.

A thorough curriculum assessment is essential to identify gaps in training that prevent CAHWs from performing their core competencies effectively. Therefore, investing in the development and implementation of curriculum and training assessments is key to complementing the recent advancements in improving and harmonizing CAHWs’ proficiency, thereby contributing to increasing the access to quality veterinary care.
